# Are sensory processing difficulties in infancy predictive of child mental health at 5‐years? Findings from the Etude Longitudinale Francaise depuis l'Enfance French national birth cohort

**DOI:** 10.1002/jcv2.70065

**Published:** 2025-11-11

**Authors:** Emma Butler, Michelle Spirtos, Mary Clarke

**Affiliations:** ^1^ Department of Population Health Royal College of Surgeons Ireland Dublin Ireland; ^2^ Discipline of Occupational Therapy Trinity College Dublin Dublin Ireland; ^3^ Department of Psychology and Psychiatry School of Population Health & Department of Psychiatry Royal College of Surgeons Ireland Dublin Ireland

**Keywords:** child mental health, sensory processing

## Abstract

**Background:**

People with neurodevelopmental and mental health conditions have been demonstrated to have elevated rates of sensory processing (SP) difficulties in research and practice but the temporal order of this relationship is unclear.

**Methods:**

This study sought to investigate whether SP difficulties in infancy predicted mental health at 5‐years as measured by the parent‐rated Strengths and Difficulties Questionnaire. Sensory group in infancy was determined by latent class assignment from behavioural indicators proposed by patient and participant involvement. Data from 10,735 5‐year‐olds from a French birth cohort, recruited at birth were analysed using regression techniques.

**Results:**

Approximately 1 in 10 infants experienced ‘definite’ sensory difficulties This group had significantly higher rates of clinical mental health symptoms at 5‐years (14.8% compared to 5.1% and 5.5% in the ‘typical’ and ‘possible’ sensory groups respectively) *x* ^2^ = 166.35, *p* ≤ 0.001. In fully‐adjusted models (controlling for cumulative sociodemographic risk, sex, history of maternal psychological difficulties), the odds of being in the group experiencing clinical levels of mental health symptoms at 5‐years increased by odds ratio 2.9 (95% confidence interval: 2.4–3.6) for children in the ‘definite’ sensory difficulties group compared to the ‘typical’ sensory group. There was no significant difference in odds between the ‘typical’ and ‘possible’ sensory groups.

**Conclusion:**

Infants with significant sensory difficulties have much higher rates of mental health symptoms by 5‐years. Sensory difficulties may be prognostic of later child mental health and thus should be addressed in early intervention. Future research in child mental health should include standardised measures of SP as a potential transdiagnostic marker for preventative intervention.

## Key Points

**What is known?**

- Sensory processing (SP) difficulties are a transdiagnostic marker of mental health in adolescence and adulthood.

**What is new?**

- SP difficulties in infancy are *predictive* of child mental health at 5‐years.

**What is relevant?**

- In practice, infants experiencing SP difficulties regardless of diagnostic status should be given greater priority for early intervention due to the relationship with later mental health.
- In research, longitudinal studies should include a standardised sensory measure of SP to elucidate the mechanism of this relationship.

## INTRODUCTION

Sensory processing (SP) refers to the way an individual integrates sensory information and manages adaptive responses to the sensory environment to engage in meaningful daily life activities (Johnson‐Ecker & Parham, 2000). SP difficulties are reported in neurodevelopmental disorders (Baranek et al., 1997; Ben‐Sasson, Hen, et al., 2009; Pfeiffer et al., 2015; Tomchek & Dunn, 2007), psychiatric disorders (van den Boogert et al., 2022), and youth at clinical high risk for psychosis (Parham et al., 2019). 80%–90% of children with Autism Spectrum Disorders (ASD) (Al‐Heizan et al., 2015) and 60% of children with Attention Deficit Hyperactivity Disorder (ADHD) (Ahn et al., 2004) experience sensory problems but children with other diagnoses also report sensory challenges (van der Linde et al., 2013). The incidence of difficulty in the regulation of responses to sensory stimuli has been suggested to be between 40% and 80% in children with disabilities and 5%–16% in typically developing peers (Critz et al., 2015; Galiana‐Simal et al., 2020; Gomez et al., 2017) depending on study methodology and sample characteristics (Ahn et al., 2004; Baranek et al., 1997; Ben‐Sasson et al., 2009; Ben‐Sasson, Hen, et al., 2009; Gouze et al., 2009; Tomchek & Dunn, 2007). The most common reported difficulties are sensory over‐responsivity (SOR) and under‐responsivity. SOR manifests as intense over‐reactivity (in terms of frequency, intensity, duration and distress/impairment) to everyday sensory inputs (Lewin et al., 2014) and SOR is described as the most prevalent sensory disorder of childhood (Reynolds & Lane, 2008). Sensory seeking has also been associated with worse mental health (van den Boogert et al., 2022). SP difficulties are associated with disruption of family routines, difficulties with self‐care, decreased play skills and social participation (Gourley et al., 2013), behavioural problems (Dean et al., 2018; Gigliotti et al., 2024), mental health problems (Carter et al., 2011; Gouze et al., 2009; Lane et al., 2010; Rossow et al., 2021), and social‐emotional difficulties (Ben‐Sasson, Carter, et al., 2009; James et al., 2011).

The relationship between increasing sensory problems associating with increasing mental health difficulties has been replicated in early childhood (Gigliotti et al., 2024; Gourley et al., 2013), middle childhood (Ben‐Sasson, Carter, et al., 2009; Dean et al., 2018; Lane et al., 2010) and adolescence/adulthood (van den Boogert et al., 2022). However, most studies involve small clinical samples (*n* < 200) and rely on cross‐sectional design.

In autistic children, Green and Ben‐Sasson (2010) proposed that SOR could cause anxiety, anxiety could cause SOR or a common latent factor could cause both SOR and anxiety (Green & Ben‐Sasson, 2010), but SP difficulties are not unique to ASD (Little et al., 2017) and similar lines of inquiry have been considered in Obsessive Compulsive Disorder (Cervin, 2023). In a birth cohort of 338 7 to 10‐year‐olds, SOR existed both independently of, and co‐morbidly with a Diagnostic and Statistical Manual of mental disorders diagnosis (Carter et al., 2011). Similarly, in a community sample of 391 four‐year‐olds, depending on criteria used, 33%–63% of children with sensory problems also had a psychiatric disorder (ADHD, Oppositional Defiance Disorder, anxiety or depression) whilst 37%–67% had sensory problems independent of a co‐morbid psychiatric disorder at that time‐point (Gouze et al., 2009). Smaller numbers of typically developing children (3%–16%) report SP difficulties (Ahn et al., 2004; Ben‐Sasson, Carter, et al., 2009; Gouze et al., 2009) but it is unclear whether they continue to develop along a typical trajectory over time. Most research has focused on the relationship between SOR and anxiety (Carpenter et al., 2019; Green & Ben‐Sasson, 2010; Green et al., 2012; Verhulst et al., 2022) but it appears that the potential association with mental health is much broader than just anxiety (van den Boogert et al., 2022). For example, sensory difficulties (beyond and including SOR) have been shown in children and young people with internalising problems (anxiety and depression) (Kotsiris et al., 2020) and externalising problems (Ben‐Sasson, Carter, et al., 2009), stress in adults (Harrold et al., 2024) and numerous adult mental health conditions (van den Boogert et al., 2022). This consistent association between SP and mental health symptoms led to ‘sensorimotor’ being added as a domain of interest in the National Institute of Mental Health's Research Domain Criteria framework in 2019 (Harrison et al., 2019).

The over‐reliance on cross‐sectional design (Gigliotti et al., 2024; Gouze et al., 2009) limits advancing the field in determining whether SP difficulties are a *risk* factor for later psychopathology and thus the temporal order of the aforementioned associations cannot be established. Three notable exceptions are; in a non‐clinical representative sample (*n* = 191), SOR in infancy predicted anxiety in pre‐school with the relationship being specific and unidirectional (Carpenter et al., 2019), in an autistic clinical sample (*n* = 149), SOR emerged earlier than, and predicted, later anxiety (Green et al., 2012) and in a transdiagnostic adult sample (*n* = 231), childhood sensory difficulties associated with lifetime anxiety diagnoses, with emotional regulation difficulties fully mediating the relationship (McMahon et al., 2019).

Another methodological limitation in the field is that most studies use standardised or non‐standardised measures of SP based on parent‐report of behavioural observations in addition to parent report of child mental health which may impact validity. SOR and anxiety can be difficult to distinguish and their physiological and behavioural symptoms can overlap (Green & Ben‐Sasson, 2010). There is also overlap between SP characteristics and dimensions of temperament (Brock et al., 2012; Nakagawa et al., 2013), the examination of individual differences in reactivity, that is, the responsiveness of emotional and arousal systems to sensory stimuli is a common element in temperament assessment (Kagan, 1997; Rothbart, 2007). SP is a neurological function whilst temperament is a broader term encompassing behavioural and personality characteristics. Ben‐Sasson et al. (2007) found that Occupational Therapists tended to rate behavioural items as representing SOR whilst psychologists tended to rate the same items as representing anxiety (Ben‐Sasson et al., 2007). In order to address these potential nomenclature limitations, He et al. (2023) have proposed a taxonomy for describing and referring to different sensory features based on five hierarchical levels of potential measurement in future research, they are sensory‐related neural excitability, perceptual sensitivity, physiological reactivity to sensory input, affective reactivity to sensory input and behavioural responsivity to sensory input (He et al., 2023). In this framework, SOR based on parent observations would be considered at the levels of behavioural or affective responsivity to sensory input.

We aimed to investigate the relationship between SP and mental health longitudinally by examining whether SP difficulties in infancy *predicted* the child's later mental health at 5‐years using a large population‐representative sample to overcome the current limitations of small clinical cross‐sectional samples. Our specific aims were: (1) to establish whether there is an association between SP in infancy and mental health at 5‐years in the general population; (2) to examine whether infant SP predicted mental health at 5‐years; (3) to examine whether infant SP predicted *clinical* levels of mental ill‐health symptoms at 5‐years. Aim 2 and 3 distinguish between a statistically significant relationship and a clinically meaningful relationship.

## MATERIALS AND METHODS

### Study design

Data was obtained from Etude Longitudinale Francaise depuis l'Enfance (ELFE), a prospective French birth‐cohort study. Details of the design, sample and measures used are presented elsewhere (Charles et al., 2020). In brief, the objective of ELFE is to study determinants of child development, health and socialisation from birth to adulthood. 349 maternity units were randomly selected nationwide, and babies were recruited from the 320 maternity units that agreed to take part during 25 selected days throughout 2011. Inclusion criteria were single/twin live births at ≥33‐week's gestation, mother ≥18‐years old, no plan to leave France within 3‐years and informed consent signed by the parents or the mother alone, with the father informed of his right to deny consent. *>*96% of the mothers who satisfied the first two inclusion criteria (*n* = 37,494) were contacted by the ELFE team during their stay in the maternity unit and 51% (18,040) agreed to participate in the cohort. The women gave birth to 18,329 babies, including 289 twin‐pairs and the children are continually followed‐up at multiple time‐points. This study uses information gathered at 1‐year old and five‐years‐old.

### Measures

#### Sensory group at 1‐year‐old

Sensory processing measures are typically not included in population‐level prospective cohort studies. Based on our previous work (Butler et al., 2025), using public and patient involvement and latent class analysis (LCA), we derived three sensory groups in infancy based on the sensory patterns of 10 parent‐reported behavioural indicators at 1‐year‐old. These three groups represented ‘typical’ SP, ‘possible’ sensory difficulties and ‘definite’ sensory difficulties. Infants were assigned to their sensory group based on their highest predicted probability. For each of the three groups the Average Posterior Probability was ≥0.8 which indicates well‐separated groups but known overlap is inherent to LCA methods.

The ‘typical’ group were easy to calm (97%), adaptable (87.6%), had low anxiety (3.1%) with no feeding concerns (1.4%). The ‘possible’ group were similar to the ‘typical’ group apart from being less adaptable (79.4%) with increased sleep difficulties (17.6%). The ‘definite’ group were the least easy to calm (68.2%), least accepting of confined spaces (71.5%), least adaptable (64.8%) and most anxious (18.4%). They exhibited more sleeping (41.8%) and feeding problems (5.1%) (see Figure S1 and Table S1 for more specific details). This ‘definite’ difficulties group was reflective of being sensory over‐responsive. See (Butler et al., 2025) for more detailed information pertaining to the development of the sensory groups.

### Covariates

#### Cumulative sociodemographic risk

Lower socioeconomic status has been found to associate with sensory difficulties (Ben‐Sasson, Carter, et al., 2009; Gouze et al., 2009) and mental health problems (Reiss et al., 2019) therefore we added a cumulative sociodemographic risk variable comprising maternal age, maternal education level, family income quintile, migrancy and maternal relationship status as a covariate. See Butler et al. (2025) for detailed information pertaining to the construction of this cumulative sociodemographic risk variable and Table S2 for sample distribution.

#### Child sex

There is conflicting evidence as to whether there are sex differences in relation to the prevalence of sensory problems (Butler et al., 2025; Gigliotti et al., 2024; Gouze et al., 2009) and known sex differences in relation to mental health (Breslau et al., 2017; Dhamala et al., 2023) thus child sex was also a covariate.

#### Maternal mental health

Parental mental health has been shown to be implicated in the transmission of psychopathology. Additionally, as the mother reported both the sensory behaviours in infancy and the Strengths and Difficulties Questionnaire (SDQ) responses, it was important to adjust for the potential contributing factor of their own mental health in the rating of the observations of their child. We included a binary yes/no variable of ‘mother experienced psychological difficulties prior to pregnancy’ as a covariate.

### Outcome

The SDQ was completed by the caregiver when the child was 5‐years‐old, as a measure of child mental health problems (A. Goodman & Goodman, 2009). The SDQ is a valid and reliable instrument to screen for emotional and behavioural problems in children aged 3–16 years and is widely used in research and clinical practice (Dachew et al., 2023). It is a parent‐rated questionnaire containing 25‐items on a 3‐point likert scale (0 = not true; 1 = somewhat true; 2 = certainly true), five items are reverse scored. Item scores are aggregated into 5 subscales. The first four subscales (emotional problems, hyperactivity/inattention, peer and conduct problems) combine to calculate a total score ranging from 0 to 40. Higher scores indicate higher difficulties. Children with higher total difficulty scores have successively higher probabilities of clinical disorder (Goodman & Goodman, 2009). Therefore, the SDQ‐total was categorised as per recommended cut‐offs (sdqinfo.org) into four categories (similar to most people, slightly raised, high and very high). Additionally, the SDQ‐total was also dichotomised at the recommended cut‐off of 17 or above representing children experiencing symptomology in the clinical range. Parent‐reported SDQ‐total scale has higher internal consistency (Cronbach's‐alpha = 0.82) and test–retest reliability than the four subscales (R. Goodman, 2001).

### Data analysis

Data analysis was conducted using STATA v.17 (StataCorp, 2021). To determine whether there was an association between clinical mental health problems at 5‐years with SP difficulties in infancy, chi‐square tests were conducted. Tests to determine whether the outcome was normally distributed were carried out. To establish whether sensory group independently predicted SDQ‐total score, multiple linear regression, adjusting for sex, sociodemographic risk and history of maternal mental health difficulties was conducted. This was repeated using logistic regression for the dichotomised clinical outcome to ascertain if sensory difficulties predicted clinically meaningful differences.

### Participants included in analysis

Of the original 18,329 babies, 4090 had withdrawn or did not take part at 1‐year follow up and 73 did not have all 10 indicators available to be assigned a sensory group at 1‐year. 11,248 children took part at 5‐years and thus had outcome information. 10,735 had all covariates of interest (Figure S2).

### Missing data

We compared characteristics of participants included in our analysis (*n* = 10,735) to those excluded due to withdrawal or missing outcome or missing independent variable data (*n* = 7594) on all variables of interest (Table S3).

### Sensitivity analyses

We conducted a sensitivity analysis applying study weights to the regression models to counteract attrition. We replicated the regression models but including each of the 10 individual LCA indicators rather than the LCA classes to ensure that there wasn't one particular indicator, such as anxiety, driving the association.

## RESULTS

Excluded individuals experienced higher levels of sociodemographic risk, sensory difficulties, preterm births, time in a neonatal intensive care unit (NICU) and total‐SDQ scores but lower levels of history of maternal mental health difficulties (Table S3).

84.4% of the sample were in the ‘typical’ SP group, 5.1% in the ‘possible’ sensory difficulties group and 10.6% in the ‘definite’ sensory difficulties group (Table 1). Children that exhibited ‘definite’ sensory difficulties at 1‐year‐old had higher mean total‐SDQ scores at 5‐years (10.8 compared to 8.1 for both ‘typical’ and ‘possible’ children) and as a result had almost three‐times higher rates of clinical levels of mental health symptoms at 5‐years (14.8% compared to 5.1% for typical and 5.5% for possible) (Pearson *χ* ^2^(2) = 166.35, *p* ≤ 0.001, Cramers *V* = 0.13). This was not merely due to higher rates in the highest SDQ category but across all SDQ‐groups (the ‘definite’ sensory difficulties group had almost three‐times higher rates in all SDQ‐groups: slightly raised (14.4% compared to 7.3% for typical and 5.9% for possible), high (8.6% compared to 3.1% for typical and 3.5% for possible), and very high (6.2% compared to 2.0% for both typical and possible) (Pearson *χ* ^2^(6) = 257.1, *p* ≤ 0.001, Cramers *V* = 0.11). There was no meaningful difference between the three sensory groups in terms of gestational age, birthweight, preterm birth, need for NICU, infant sex or history of maternal psychological difficulties. Both ‘possible’ and ‘definite’ difficulties groups reported higher rates of cumulative sociodemographic risk compared to the ‘typical’ group. 7.2% of ‘definite’ and 5.9% of ‘possible’ reported high sociodemographic risk compared to only 2.1% in the ‘typical’ group. Pearson *χ* ^2^(6) = 239.8, *p* ≤ 0.001, Cramers *V* = 0.11).

**TABLE 1:** Sample characteristics by infant sensory processing groups.

	Total (*n* = 10,735)	Typical 84.4% (*n* = 9057)	Possible 5.1% (*n* = 542)	Definite 10.6% (*n* = 1136)
Clinical level on SDQ^a^ (%yes)	6.1	5.1	5.5	14.8
SDQ categories				
Close to average	85.9	87.6	88.6	70.9
Slightly raised	8.0	7.3	5.9	14.4
High	3.7	3.1	3.5	8.6
Very high	2.4	2.0	2.0	6.2
SDQ‐total (*M* ^b^, SD^c^)	8.4 (4.7)	8.1 (4.6)	8.1 (4.7)	10.8 (5.4)
Min–max	0–33	0–33	0–30	0–33
Gestational age (week) (Med^d^, IQR^e^) (*n* = 10,653)	39 (39–40)	39 (39–40)	39 (38–40)	39 (38–40)
Birthweight (g) (*M*, SD) (*n* = 10,593)	3341 (480)	3347 (479)	3341 (452)	3294 (497)
NICU^f^ (% yes) (*n* = 9320)	5.7	5.5	7.5	6.5
Preterm (<37‐weeks) (% yes) (*n* = 10,653)	4.3	4.2	4.8	5.1
Child sex (% male)	50.9	50.8	49.6	52.0
History of maternal psychological difficulties (% yes)	26.2	26.3	23.4	27.3
Cumulative sociodemographic risk^g^				
None	45.0	47.3	33.2	32.0
Low	31.8	31.6	31.6	33.2
Moderate	20.4	19.0	29.3	27.6
High	2.9	2.1	5.9	7.2

Abbreviation: IQR, interquartile range.

^a^ Strengths and Difficulties Questionnaire.

^b^ Mean.

^c^ Standard deviation.

^d^ Median.

^e^ Interquartile range.

^f^ Neonatal Intensive Care Unit.

^g^ Cumulative variable comprised of maternal age at birth, maternal education level, maternal relationship status, migrancy and family income quintile.

In the multiple linear regression model, adjusting for child sex, cumulative sociodemographic risk and history of maternal psychological difficulties, sensory group, specifically the ‘definite’ sensory difficulties group remained a significant predictor of total‐SDQ (Table 2).

**TABLE 2:** Linear regression model for sensory group on SDQ‐total score with adjustment for covariates (*n* = 10,735).

Fully‐adjusted linear regression model (*n* = 10,735)			
	Co‐efficient (95% CI^a^)	Std. error	*t*
‘Typical’ sensory processing—Base			
‘Possible’ difficulties	−0.21 (−0.61 to 0.19)	0.20	−1.04
‘Definite’ difficulties	2.44 (2.16–2.73)	0.15	16.80
Sex—Male base			
Female	−1.05 (−1.22 to −0.87)	0.09	−11.84
Cumulative sociodemographic risk^b^—None base			
Low	0.64 (0.44–0.84)	0.10	6.21
Moderate	1.61 (1.38–1.84)	0.12	13.54
High	2.05 (1.51–2.58)	0.27	7.53
History of maternal psychological difficulties—No base			
Yes	0.89 (0.69–1.09)	0.10	8.86

*Note:* Adjusted for sex, cumulative sociodemographic risk and history of maternal psychological difficulties.

^a^ Confidence interval.

^b^ Cumulative variable comprised of maternal age at birth, maternal education level, maternal relationship status, migrancy and family income quintile.

This linear model indicated that the combined explanatory variables have a statistically significant relationship with SDQ‐total, explaining 6.7% of the variance in SDQ‐score. The coefficients show the average change in SDQ‐total that is associated with a 1‐unit increase in that explanatory variable. ‘Definite’ sensory difficulties had the largest coefficient change 2.4, assuming the other variables are held constant. As all of the explanatory variables except for ‘possible’ sensory difficulties have a statistically significant relationship with the total‐SDQ, we examined the standardised regression co‐efficients (beta) in order to compare the strength of the coefficients to each other (Table S4), this confirmed that ‘definite’ sensory difficulties at 1‐year had the strongest effect on the outcome.

**FIGURE 1:**
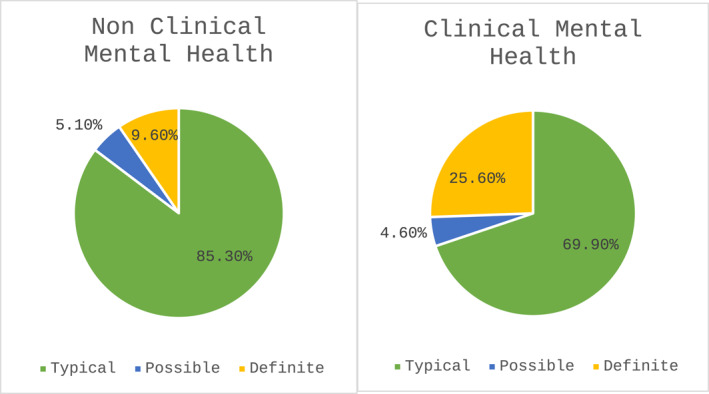
Pie‐charts showing the proportion of children from the infant sensory processing groups in the non‐clinical and clinical range of mental health symptoms as measured by the SDQ‐total at 5‐years (*n* = 10,735).

There was a 2.7 SDQ points difference between ‘definite’ difficulties group and the other two groups. To explore if this is a clinically meaningful difference, we examined the proportion of sensory groups in the clinical and non‐clinical range (Figure 1) and conducted a logistic regression with the outcome being a binary variable of clinical levels of SDQ‐symptoms (Table 3). Whilst there were similar proportions of children from the ‘possible’ sensory group in both the clinical and non‐clinical mental health groups at 5‐years, there was a much lower proportion of children from the ‘typical’ group and a much higher proportion of children from the ‘definite’ group in the clinical range group at 5‐years compared to the non‐clinical mental health group (Figure 1).

**TABLE 3:** Logistic regression models for sensory group on clinical levels of mental health symptoms at 5‐years as measured by dichotomised SDQ‐total (>16) with adjustment for covariates (*n* = 10,735).

Fully‐adjusted logistic regression model (*n* = 10,735)			
	Odds ratio (95% CI^a^)	Std. error	*z*
‘Typical’ sensory processing—Base			
‘Possible’ difficulties	1.00 (0.69–1.45)	0.19	0.01
‘Definite’ difficulties	2.92 (2.39–3.58)	0.30	10.39
Sex—Male base			
Female	0.58 (0.49–0.69)	0.05	−6.14
Cumulative sociodemographic risk^b^—None base			
Low	1.44 (1.17–1.77)	0.15	3.42
Moderate	2.04 (1.62–2.58)	0.25	5.96
High	2.72 (1.83–4.04)	0.55	4.94
History of maternal psychological difficulties—No base			
Yes	1.78 (1.48–2.13)	0.16	6.21

*Note:* Adjusted for sex, cumulative sociodemographic risk and history of maternal psychological difficulties.

^a^ Confidence interval.

^b^ Cumulative variable comprised of maternal age at birth, maternal education level, maternal relationship status, migrancy and family income quintile.

In the adjusted logistic model, the odds of being in the clinical group at 5‐years increased 2.9 times (95% CI: 2.4–3.6) for those in the ‘definite’ sensory difficulties group compared to the ‘typical’ group. The predicted probability of experiencing a clinical level of mental health difficulties at 5‐years was 5.2% (95% CI: 4.7%–5.6%) for children who had ‘typical’ sensory at 1‐year, 5.2% (95% CI: 3.4%–6.9%) for children who had ‘possible’ sensory difficulties and 13.5% (95% CI: 11.5%–15.5%) for children who had ‘definite’ sensory difficulties from the logistic model (Figure S3). As per Figure S3. The predicted probability of clinical levels of mental health symptoms at 5‐years for the ‘definite’ group is higher than the average prevalence for 5 to 10‐year‐olds (9.5%) in 2017 (NHS Digital, 2018).

The sensitivity analyses (Table S5a,b) showed that applying weights to adjust for attrition did not substantially change the findings. Additionally, 8 of the 10 LCA indicators had a significant association with outcome highlighting that there wasn't one particular characteristic that could potentially have been considered to be a marker of temperament or anxiety driving the association (Table S6a,b).

## DISCUSSION

We examined the relationship between sensory difficulties in infancy and mental health at 5‐years and whether the child's SP at 1‐year predicted their mental health at 5‐years. We found that children with ‘definite’ sensory difficulties had significantly worse mental health at 5‐years than the infants who had ‘typical’ or ‘possible’ sensory difficulties. A meta‐analysis (van den Boogert et al., 2022) showing large to very large effect sizes for the association between sensory differences and many psychiatric conditions, concluded that SP difficulties can be considered as a non‐specific transdiagnostic phenotype associated with a broad spectrum of psychiatric conditions in adolescents and adults. However, the temporal order of this relationship could not be addressed. We have added to the body of knowledge by highlighting that in children, SP difficulties in infancy were predictive of mental health broadly at 5‐years even after adjusting for child sex, cumulative sociodemographic risk and history of maternal psychological difficulties.

Our findings concur with other studies (Ben‐Sasson, Carter, et al., 2009; Dean et al., 2018; Gigliotti et al., 2024; Gourley et al., 2013; Rossow et al., 2021), that is, that children with SP difficulties have a significantly higher rate of mental health difficulties or worse symptom severity (Cervin, 2023). However, our study significantly contributes to this body of knowledge by providing evidence that these sensory difficulties *precede* mental health difficulties and could be considered prognostic of later mental health difficulties. This finding is supported by Carpenter et al. (2019) who demonstrated in a representative sample that SOR at 2‐years predicted anxiety at 6‐years and the relationship was unidirectional (Carpenter et al., 2019). Similarly, smaller clinical, mainly adult samples have also provided evidence that SOR precedes anxiety (Green et al., 2012; McMahon et al., 2019; Verhulst et al., 2022). A limitation of one study is that sensory problems in childhood were retrospectively reported and this recall bias could have influenced the findings (McMahon et al., 2019). Here we demonstrated that infants with prospectively measured SP difficulties had significantly higher rates of clinical levels of mental health symptoms at 5‐years.

In support of previous evidence (Dean et al., 2022), our findings confirm that SP difficulties are predictive of later poor mental health but whether the relationship is causal in nature cannot be determined. As per Green and Ben‐Sasson (2010) SOR could cause anxiety, anxiety could cause SOR, or both SOR and anxiety could have a common underlying cause (Green & Ben‐Sasson, 2010) such as genetic effects (Van Hulle et al., 2012). Future research could examine if there is a difference in the provision of early intervention between those children who experienced SP difficulties in infancy who later did or did not display poor mental health at 5‐years. Additionally, these children should be followed longitudinally as it is also possible that the infants who experienced SP difficulties in infancy who did not display poor mental health at 5‐years, may display poor mental health later in the life‐course, for example, during adolescence when protective factors may change.

Most sensory research has been conducted in small clinical samples or diagnostic groups, which likely leads to inflated prevalence rates compared to population‐samples. There is a paucity of research on the extent to which children in the general population may show particular sensory patterns (Dean et al., 2022) and whether the relationship between sensory patterns and mental health holds in the typical population. In line with previous studies (Carpenter et al., 2019; Dean et al., 2022; Little et al., 2017), we used a population‐based sample so that the sensory profile of *all* children, regardless of diagnoses, would be considered. We found that 15.6% of infants had ‘possible’ or ‘definite’ sensory problems. Another study using a representative sample of 191 two‐year‐olds, using 9 sensory experiences as a measure of SP found that 20% of parents reported their child experienced SOR in at least one sensory domain (Carpenter et al., 2019). This study and ours both provide evidence that SOR predicted later mental health.

The scale or pattern of sensory differences may also be important (Fulkerson, 2014), again highlighting the need for a unified taxonomy of measurement of sensory constructs (He et al., 2023). We found that there was no difference in mental health outcomes at 5‐years between infants who had ‘typical’ SP and those with ‘possible’ sensory difficulties. This may point to a threshold effect, that is, having one or two sensory issues is a normal variation of typical child development and does not indicate an underlying disorder or reason for concern. However, infants who are experiencing *multiple* sensory difficulties impacting many facets of their day‐to‐day life such as feeding, sleeping, play are at greater risk and need to be prioritised for early intervention regardless of whether they are presenting with a known diagnosis. Alternatively, the lack of association with the ‘possible’ class could be attributable to reduced statistical power to detect a difference as the ‘possible’ group was much smaller than the ‘definite’ group.

Therapists and researchers often overlook the influences of sensory experiences on behavioural and affective regulation (Van Hulle et al., 2018). Children experiencing sensory difficulties are typically referred to paediatric occupational therapy (OT) (O'Donoghue et al., 2021). Occupational therapists are dual‐trained in physical and mental health and can play a major role in identifying and intervening with this group of high‐risk children earlier in the life‐course.

Sensory research at population‐level is restricted by the absence of standardised sensory measures used in prospective cohort studies and thus SP is an overlooked area in child development research. Future longitudinal cohort studies should strongly consider including a standardised measure of SP so that its prognostic ability as a transdiagnostic marker can be investigated further. Furthermore, all of the studies cited here, including ours, apart from (Lane et al., 2010) rely on parent or self‐reported questionnaires pertaining to both sensory symptoms and mental health. The overlap between SOR and anxiety symptoms (Ben‐Sasson et al., 2007) compromises the attempts to study these associations using parent‐report measures. Teasing out the independence of SP difficulties, or the contribution of SP to individual differences in temperament, is also of importance. Therefore future research should consider other levels of measurement of sensory differences, beyond behaviour, as per the hierarchical taxonomy proposed by (He et al., 2023). Of interest, the one study cited using an objective measure demonstrated a correlation between electrodermal responses and SOR (Lane et al., 2010), this demonstrates an association at the physiological reactivity to sensory input level (He et al., 2023).

### Strengths and limitations

The use of a large, population based prospective longitudinal sample is a significant strength of this study. Using the LCA sensory groups could have impacted the validity of the findings, however no standardised sensory measures were available in longitudinal samples. There is known uncertainty in class assignment that comes with LCA and this may have biased the relationships in the regression analyses. The authors acknowledge that dependent on your discipline lens, our sensory groups could be considered characteristic of temperament or anxiety rather than sensory specific, but this overlap is a known problem in the field of sensory research (Ben‐Sasson et al., 2007; Brock et al., 2012). However, our patient and participant involvement (PPI) group who selected the sensory indicators consisted of parents of children with SP difficulties whose qualifications spanned nursing, OT, speech and language therapy and audiology, thereby not limiting perspectives to any one discipline. Irritability, food selectivity and sleep have been found to associate with SOR and anxiety (Carpenter et al., 2019; Thomas et al., 2015). Our sensitivity analysis (Table S6a,b) highlighted that 8 of the 10 sensory indicators associated with outcome and so it was not just the anxiety or sleep indicators driving the association.

We did not have information available as to whether the child had a specific diagnosis and thus we could not examine whether the definite sensory difficulties group was completely comprised of children with known diagnoses. However as children move within and across diagnostic categories (Healy et al., 2022; Morales et al., 2023) and there is great heterogeneity within diagnostic groups (Caspi et al., 2020), it is important to consider the child's symptoms/presentations regardless of their diagnoses.

Furthermore, similar to the other studies cited here, as it was the primary caregiver reporting both the sensory behaviours and the SDQ, this could inflate associations. Future research could consider objective measures of SP at different levels of analysis (He et al., 2023) and/or triangulating behavioural observations from multiple informants, such as the child, the caregiver and their childcare provider. Further, one potential explanation for the association with SOR and anxiety could be overlapping genetic risk (Van Hulle et al., 2012), adjusting for maternal history of mental health in our analysis is a first step in teasing this out.

Like all longitudinal studies there was selective attrition. Our sensitivity analysis with study weights applied to counteract attrition provides reassurance as it did not meaningfully alter the findings (Table S5a,b).

### Conclusion

Infants experiencing sensory difficulties have significantly higher rates of mental health symptoms by 5‐years. Sensory problems in infancy could be used as an early indicator of later mental health difficulties with potential usefulness for informing interventions. Longitudinal cohort studies should include measures of SP at different hierarchical levels to ascertain its prognostic potential and in practice standardised sensory assessments should be conducted regularly in order to identify children at‐risk of poor mental health earlier.

## AUTHOR CONTRIBUTIONS

**Emma Butler**: Conceptualization; data curation; formal analysis; funding acquisition; methodology; software; visualization; writing—original draft; writing—review and editing. **Michelle Spirtos**: Methodology; supervision; writing—review and editing. **Mary Clarke**: Conceptualization; methodology; supervision; writing—review and editing.

## CONFLICT OF INTEREST STATEMENT

The authors declare no conflicts of interest.

## ETHICAL CONSIDERATIONS

This secondary analysis was approved on 12/01/2023 by the Research Ethics committee for the Royal College of Surgeons Ireland (RCSI RIMS 212610659). For the original ELFE study, ethical approvals for data collection in maternity units and for each data collection wave during follow‐up were obtained from the national advisory committee on information processing in health research (CCTIRS: Comité Consultatif sur le Traitement de l’Information en matiére de Recherche dans le domaine de la Santé), the national data protection authority (CNIL: Comission Nationale Informatique et Liberté) and, in case of invasive data collection such as biological sampling, the committee for protection of persons engaged in research (CPP: Comité de Protection des Personnes). The ELFE study was also approved by the national committee for statistical information (CNIS: Conseil National de l’Information Statistique). Informed consent signed by the parents or the mother alone was secured, with the father being informed of his right to deny consent for participation (Charles et al., 2020).

## Supporting information

Supporting Information S1

## Data Availability

Data is available upon request from https://plateforme‐acces‐donnees‐elfe‐france.site.ined.fr.
