# Supplementary material for: Are sensory processing difficulties in infancy predictive of child mental health at 5‐years? Findings from the Etude Longitudinale Francaise depuis l'Enfance French national birth cohort
Source: JCPP Adv. 2025 Nov 11:e70065. Online ahead of print. doi: 10.1002/jcv2.70065 (PMC13337138; doi:10.1002/jcv2.70065)
Supplement: Supplementary file 1 — Supporting Information S1 [file JCV2-9999-e70065-s001.docx]

**Are sensory processing difficulties in infancy predictive of child mental health at 5-years? Findings from the ELFE French national birth cohort**

**Supporting Information**

**Fig S1.** Distribution of the ten behavioural indicators across the three latent sensory groups


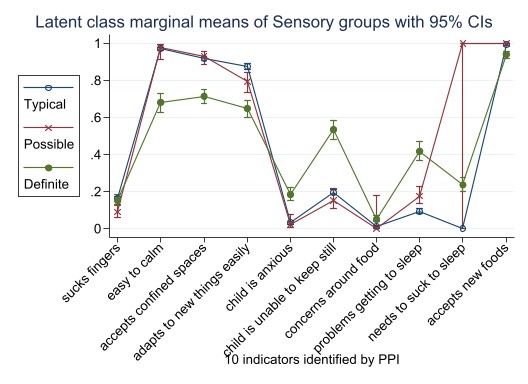


**Table S1.** Estimated proportion of sample affirming each indicator in each class in 3-class LCA solution (n=14,166 in the original study)

| Indicator | **Typical (n~10086)** | **Possible (n~1063)** | **Definite (n~3017)** |
| --- | --- | --- | --- |
| Sucks fingers | 16.4% | 9.0% | 15.3% |
| Easy to calm | 97% | 97.6% | 68.2% |
| Accepts confined spaces | 91.7% | 93% | 71.5% |
| Adapts to new situations | 87.6% | 79.4% | 64.8% |
| Child is anxious | 3.1% | 2.3% | 18.4% |
| Unable to keep still | 19.7% | 15.3% | 53.4% |
| Concerns about food | 1.4% | .21% | 5.1% |
| Problems going to sleep | 9.4% | 17.6% | 41.8% |
| Sucks to sleep | <.01% | 100% | 23.6% |
| Accepts new foods | 99.5% | 100% | 94% |

**Table S2.** Distribution of the individual sociodemographic indicators contributing to the cumulative sociodemographic groups

| N=10735 | No risk – assign 0  (%) | Low risk – assign 1  (%) | Moderate risk – assign 2 (%) | High risk – assign 3 (%) |
| --- | --- | --- | --- | --- |
| Maternal Migrancy | 92.2 | 7.8 |  |  |
| Maternal age | 71.8 | 28.2 |  |  |
| Maternal relationship | 97.3 | 2.7 |  |  |
| Maternal education level | 44.6 | 24.1 | 27.3 | 4.0 |
| Family income quintile | 50.1  (quintile 1+2) | 18.1  (quintile 3) | 31.8  (quintile 4+5) |  |
| Cumulative sociodemographic risk | 45.0 | 31.8 | 20.4 | 2.9 |

**Fig S2.** Participant flow-diagram

18329 recruited at birth

4090 did not participate, 73 missing some sensory indicators @1-year

14166 had sensory group at 1-y

11248 took part at 5-years, 394 did not have the sensory information

10854 participated at 5-y & had sensory group at 1-y

90 missing sex, 29 missing maternal mental health

10735 had all covariates

**Table S3.** Comparison of characteristics of those included (n=10,735) to those excluded (n=7594) due to withdrawal or missing outcome or missing covariate of interest

|  | Total  N=18329 | Included  N=10735 | Excluded  N=7594 |
| --- | --- | --- | --- |
| Sex (%male) | 51.1 | 50.9 | 51.8 (n=3297) |
| Cumulative sociodemographic risk (%): n=3431 | | | |
| None | 40.3 | 45.0 | 25.5 |
| Low | 31.7 | 31.8 | 31.4 |
| Mod | 23.6 | 20.4 | 33.5 |
| High | 4.5 | 2.9 | 9.6 |
| History of maternal mental health (%yes) | 24.0 | 26.2 | 16.9 (n=3282) |
| Sensory group (n=14166): n=3431 | | | |
| Typical Sensory | 82.2 | 84.4 | 75.2 |
| Possible Difficulty | 6.0 | 5.1 | 9.0 |
| Definite Difficulties | 11.9 | 10.6 | 15.8 |
| Clinical on SDQ (% yes) | 6.2 | 6.1 | 7.8 (n=513) |
| Gestational age (weeks)  MED IQR | N=13947  39 (38-40) | N=10653  39(39-40) | N=3294  39(38-40) |
| NICU (%yes) | 6.6  N=12209 | 5.7  N=9320 | 9.2  N=2889 |
| Birthweight (grams) (M, SD) | 3316.6 (492.5)  N=13859 | 3341.0 (480.0)  N=10593 | 3237.4 (523.3)  N=3266 |
| Preterm (born between 32-36+6) (% yes) | 5.3  N=13947 | 4.3  N=10653 | 8.3  N=3294 |
| SDQ-total (M, SD) | 8.4 (4.8)  N=11248 | 8.4 (4.7)  N=10735 | 8.9 (4.9)  N=513 |

**Table S4.** Beta co-efficients and standardised beta co-efficients for each independent variable on SDQ-total score in the linear multiple regression model. (n=10,735)

| Predictor | Co-efficient | Standardised Beta coefficient* |
| --- | --- | --- |
| Sensory group (typical base): | | |
| Possible difficulty | -0.211 | -0.01 |
| Definite difficulties | 2.442 | 0.16 |
| Sex (male base): | | |
| Female | -1.047 | -0.11 |
| Cumulative sociodemographic risk (none base): | | |
| Low | 0.637 | 0.06 |
| Moderate | 1.609 | 0.14 |
| High | 2.045 | 0.07 |
| History of maternal psychological difficulties (no base): | | |
| Yes | 0.892 | 0.08 |

*The standardised beta co-efficients are interpreted as: a 1-standard deviation increase in X leads to <that beta value> amount increase in predicted SDQ-total score.

**Fig S3.** The predicted probability of experiencing a clinical level of mental health symptoms at 5-years for each sensory group adjusting for sex, cumulative sociodemographic risk and maternal mental health


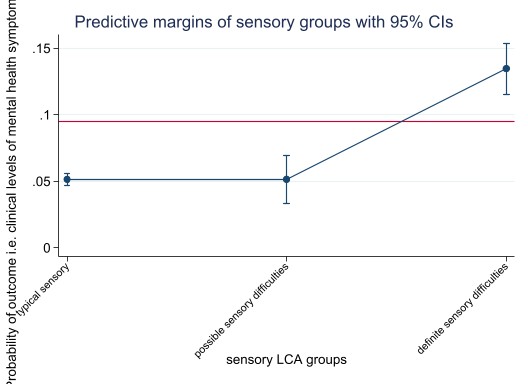


Y reference line of 9.5% is the prevalence of mental health disorders in 5-10-year-olds in 2017 (NHS Digital (2018). Mental health of children and young people in England 2017) rcpch.ac.uk

**Table S5a.** Multiple linear regression analysis but with study weights accounting for attrition applied

|  | Co-efficient (95% CI) | Std. Error | t |
| --- | --- | --- | --- |
| ‘typical’ sensory base | | | |
| ‘possible’ difficulties | -0.19 (-0.80-0.43) | 0.31 | -0.59 |
| ‘definite’ difficulties | 2.17 (1.71-2.64) | 0.24 | 9.12 |
| Sex ‘male’ base | | | |
| Female | -1.15 (-1.43- -0.87) | 0.14 | -8.07 |
| Cumulative sociodemographic risk ‘none’ base | | | |
| Low | 0.77 (0.46-1.08) | 0.16 | 4.93 |
| Moderate | 1.86 (1.54-2.17) | 0.16 | 11.63 |
| High | 1.88 (1.09-2.67) | 0.40 | 4.69 |
| History of maternal psychological difficulties ‘no’ base | | | |
| Yes | 0.97 (0.69-1.25) | 0.14 | 6.74 |

**Table S5b.** Logistic regression analysis but with study weights accounting for attrition applied

|  | Odds Ratio (95% CI) | Std. Error | z |
| --- | --- | --- | --- |
| ‘typical’ sensory base | | | |
| ‘possible’ difficulties | 0.91 (0.53-1.55) | 0.25 | -0.35 |
| ‘definite’ difficulties | 2.55 (1.94-3.36) | 0.36 | 6.68 |
| Sex ‘male’ base | | | |
| Female | 0.60 (0.47-0.76) | 0.07 | -4.29 |
| Cumulative sociodemographic risk ‘none’ base | | | |
| Low | 1.56 (1.16-2.10) | 0.24 | 2.93 |
| Moderate | 2.36 (1.78-3.12) | 0.34 | 6.04 |
| High | 2.73 (1.61-4.63) | 0.21 | 3.74 |
| History of maternal psychological difficulties ‘no’ base | | | |
| Yes | 1.73 (1.36-2.19) | 0.21 | 4.55 |

**Table S6a**. Multiple linear regression analysis but each LCA indicator instead of overall LCA groups as predictors

|  | Co-efficient (95% CI) | Std. Error | t |
| --- | --- | --- | --- |
| Sex ‘male’ base | | | |
| Female | -1.15 (-1.43- -0.87) | 0.14 | -8.07 |
| Cumulative sociodemographic risk ‘none’ base | | | |
| Low | 0.77 (0.46-1.08) | 0.16 | 4.93 |
| Moderate | 1.86 (1.54-2.17) | 0.16 | 11.63 |
| High | 1.88 (1.09-2.67) | 0.40 | 4.69 |
| History of maternal psychological difficulties ‘no’ base | | | |
| Yes | 0.97 (0.69-1.25) | 0.14 | 6.74 |
| Each individual sensory behaviour indicator: | | | |
| Sucks fingers | 0.27 (0.04-0.50) | 0.12 | 2.32 |
| Easy to calm | -1.58 (-1.92- -1.25) | 0.17 | -9.23 |
| Accepted confined | -0.58 (-0.85- - -0.31) | 0.14 | -4.23 |
| Adapts to new | -0.45 (-0.70- -0.19) | 0.13 | -3.43 |
| Child anxious | 0.84 (0.44- 1.24) | 0.20 | 4.14 |
| Unable to keep still | 1.79 (1.58-2.00) | 0.11 | 16.98 |
| Concern food | 1.93 (1.33-2.54) | 0.31 | 6.25 |
| Problems to sleep | 0.49 (0.23-0.74) | 0.13 | 3.75 |
| **Suck to sleep** | -0.03 (-0.36-0.30) | 0.17 | -0.20 |
| **Accepts new foods** | -0.04 (-0.85-0.77) | 0.41 | 0.93 |

**Table S6b.** Logistic regression analysis but each LCA indicator instead of overall LCA groups as predictors

|  | Odds Ratio (95% CI) | Std. Error | z |
| --- | --- | --- | --- |
| Sex ‘male’ base | | | |
| Female | 0.58 (0.49- 0.69) | 0.05 | -6.39 |
| Cumulative sociodemographic risk ‘none’ base | | | |
| Low | 1.40 (1.15-1.71) | 0.14 | 3.33 |
| Moderate | 1.92 (1.56-2.37) | 0.21 | 6.12 |
| High | 2.54 (1.74-3.73) | 0.50 | 4.80 |
| History of maternal psychological difficulties ‘no’ base | | | |
| Yes | 1.76 (1.48-2.08) | 0.15 | 6.50 |
| Each individual sensory behaviour indicator: | | | |
| **Sucks fingers** | 1.21 (0.98-1.50) | 0.1 | 1.77 |
| Easy to calm | 0.56 (0.45-0.71) | 0.07 | -4.81 |
| Accepted confined | 0.68 (0.55-0.84) | 0.08 | -3.50 |
| Adapts to new | 0.71 (0.57-0.87) | 0.08 | -3.26 |
| Child anxious | 1.41 (1.06-1.90) | 0.21 | 2.32 |
| Unable to keep still | 2.08 (1.75-2.47) | 0.18 | 8.43 |
| Concern food | 2.45 (1.64-3.66) | 0.50 | 4.39 |
| Problems to sleep | 1.35 (1.09-1.66) | 0.14 | 2.86 |
| **Suck to sleep** | 1.09 (0.83-1.44) | 0.15 | 0.61 |
| **Accepts new foods** | 1.52 (0.76-3.03) | 0.53 | 1.19 |
